# The role of pharmacists in developing countries: the current scenario in Pakistan

**DOI:** 10.1186/1478-4491-7-54

**Published:** 2009-07-13

**Authors:** Saira Azhar, Mohamed Azmi Hassali, Mohamed Izham Mohamed Ibrahim, Maqsood Ahmad, Imran Masood, Asrul Akmal Shafie

**Affiliations:** 1 Social and Administrative Pharmacy, School of Pharmaceutical Sciences, Universiti Sains Malaysia, Penang, Malaysia; 2 Department of Pharmacy, University of Sargodha, Punjab, Pakistan

## Abstract

During the past few years, the pharmacy profession has expanded significantly in terms of professional services delivery and now has been recognized as an important profession in the multidisciplinary provision of health care. In contrast to the situation in developed countries, pharmacists in developing countries are still underutilized and their role as health care professionals is not deemed important by either the community or other health care providers. The aim of this paper is to highlight the role of pharmacists in developing countries, particularly in Pakistan. The paper draws on the literature related to the socioeconomic and health status of Pakistan's population, along with background on the pharmacy profession in the country in the context of the current directions of health care.

The paper highlights the current scenario and portrays the pharmacy profession in Pakistan. It concludes that although the pharmacy profession in Pakistan is continuously evolving, the health care system of Pakistan has yet to recognize the pharmacist's role. This lack of recognition is due to the limited interaction of pharmacists with the public. Pharmacists in Pakistan are concerned about their present professional role in the health care system. The main problem they are facing is the shortage of pharmacists in pharmacies. Moreover, their services are focused towards management more than towards customers. For these reasons, the pharmacist's role as a health care professional is not familiar to the public.

## Review

### Background

The World Health Organization (WHO) has defined health as the state of complete physical, mental and social well-being and not merely the absence of disease or infirmity [1]. Within the context of this definition, health care providers play a major role in striving for health in a population. In terms of modern health care delivery, studies have shown that engaging multidisciplinary expertise is one of the goals for achieving ultimate population health [2]. Although the pharmacy profession is recognized for its importance as a health care provider in many developed countries, in most developing countries it is still underutilized [2].

#### The pharmacist as a health care provider

Pharmacy is the health profession that links the health sciences with the basic sciences; it is committed to ensuring the safe and effective use of medication [3]. Pharmacists' professional roles and responsibilities have evolved historically from a focus on medication compounding and dispensing to extended pharmaceutical care services [4].

An increase in health demands, with a complex range of chronic medicines and poor adherence to prescribed medicines, has forced pharmacists to take a patient-centered approach [5]. The paradigm shift for pharmacy practice took turn in 1990, when Hepler and Strand introduced the term "pharmaceutical care" [6]. Over the last few decades, pharmacy organizations and academic training programmes around the world have promoted pharmaceutical care as a philosophy and standard of provision of care for patients [7]. In essence, the pharmaceutical care concept has transformed the pharmacy profession to be more accountable in patient care, especially to ensure that a patient achieves positive outcomes from drug therapy [8].

In many parts of the world, pharmacists have played a significant role in provision of pharmaceutical care services. In addition, it is also widely believed that pharmacists can make a great contribution to the provision of the primary health care, especially in developing countries [9,10]. Their role varies in different parts of the world: some deal with the preparation and supply of medicines, while some focus on sharing pharmaceutical expertise with doctors, nurses and patients [11].

#### The pharmacy profession in the international context

WHO has contributed effectively towards encouraging and defending the role of pharmacists worldwide [9]. Although all health care providers and the public are rationally involved in using drugs, WHO has recommended a special role for pharmacists, particularly in quality assurance and the safe and effective administration of drugs[12]. The International Pharmaceutical Federation (FIP) and WHO developed the concept of "The seven star pharmacist", which stated that a well-rounded pharmacist should be a compassionate care giver, decision maker, active communicator, lifelong learner and good manager; and should possess good leadership qualities and the ability to be a teacher and researcher [13]. According to WHO, future pharmacists must possess specific knowledge, attitudes, skills and behaviors in support of their roles [14,15].

Due to the increasing demand for pharmacists in public health, WHO recommends a ratio of one pharmacist per 2000 population in order for optimal health care to be delivered. Besides their pivotal role in public health, pharmacists can also act as advisors to physicians and nurses and contribute to policy decisions [16].

#### Pharmacy practice in developing countries

Pharmacy practice models in developing countries vary significantly from one country to another. Some of the major issues identified as barriers to effective pharmacy practice models in these countries include an acute shortage of qualified pharmacists and no implementation of dispensing separation practices – especially in countries where the pharmacist is not the sole dispenser and medical practitioners are allowed to dispense as well – and a lack of standard practice guidelines.

For example, in a country such as Malaysia, which is one of the leading countries in terms of economic growth in the south-east Asia region, there is an acute shortage of pharmacists practicing in community settings [17]. Data for 2006 showed that the ratio of pharmacists to population in Malaysia was 1:6207 [18].

Doctors in Malaysia still dispense medications as a part of their professional practice. There is still no separation of functions related to drug dispensing and prescribing between doctors' clinics and pharmacies. Registered pharmacists are not the only professionals with the legal right and responsibility of dispensing medications. Although the call for separation has been made for the last 20 years, the government still believes that due to the shortage of pharmacists the separation cannot be implemented. Another reason for delaying the separation is the objection of medical practitioners [19,20].

Looking at the perspective of African nations such as Ghana, the shortage of pharmacists is even worse: it has been reported that only 619 pharmacists are serving 2.9 million people in Greater Accra [21], which is far behind the WHO recommendation (1:2000).

In developing countries, the urban population is more affluent [22]. As a result, health professionals such as pharmacists prefer to work in cities rather than rural areas [9,23]. The lack of human resources creates a significant difference between the health services available in the urban and rural areas. In many cases this is due to the shortage of pharmacists [24,25].

Other countries, such as India, have a comparatively high number of trained pharmacists, but their pharmacy training is focused more towards the industrial sector. This is due mainly to the demand from the industrial side and the focus of the national pharmacy curriculum in most universities, which covers mainly subjects pertaining to the production aspects of pharmaceuticals [24].

Pharmaceutical services in developing countries face some specific challenges unlike those faced by pharmacists in the developed world. In most developing countries, lack of appropriate and good-quality medicines is the most common problem encountered [7]. Irrational use of medicine and weak regulatory enforcement of drug sales are also serious issues in developing countries. For example, findings from a survey conducted in a rural region of Ghana revealed that drug retailers in five pharmacy shops were found to have little or no training in pharmacy; the population bought drugs without prescriptions; the staff of these shops contributed to drug misuse by providing misinformation about drugs and selling drugs according to popular demand [26].

### A brief overview of the socioeconomic and health status of Pakistan's population

Pakistan extends from the mountain valleys of the Himalayas to along the Arabian Sea bordering India, China, Afghanistan and Iran. It is strategically located along the ancient trade route between Asia and Europe[27]. In 1947, Pakistan was created as British rule came to an end in India. In 1971, East Pakistan demanded independence, and after a bloody civil war it was transformed into what is now the country of Bangladesh. As one of the most populous countries in the world, Pakistan faces enormous economic and social crises. Fortunately, however, it possesses an abundance of natural resources that can help it overcome these challenges [28].

With a population of approximately 160 million, Pakistan is the sixth most populous country in the world [29]. The average growth rate in the economy over the past five years was 7%. Pakistan has enjoyed more than five years of sound economic growth and poverty reduction since 2002, yet in 2004/05, 24% – nearly 40 million – were still living below the national poverty line[30]. In 2004/05, 52% of five to nine-year-olds went to school.

Life expectancy is 64 years for men and 66 for women; 50% of the adult population is illiterate. One in 10 children dies before their fifth birthday. Every year 25 000–30 000 women die from complications of pregnancy and childbirth. There are an estimated 87 000 people living with HIV in Pakistan. In 2004/05, 66% of the population of Pakistan had access to a tap or hand water pump[31].

According to the adjusted gross domestic product (GDP), the per capita income comes to USD 812 in 2006 [32]. Poverty rates, which had fallen substantially in the 1980s and early 1990s, started to rise again towards the end of the decade. More importantly, differences in income per capita across regions have persisted or increased. Poverty varies significantly between rural and urban areas and from province to province, from a low of 14% in urban Sindh Province to 41% in the rural North Western Frontier Province (NWFP) [33]. Pakistan still faces formidable challenges (political, attitudinal and policy) to fully develop human capital, improve investment and increase productivity by bringing the economy to a rate achieved in earlier decades, i.e. an annual growth of 5% or more, to significantly reduce poverty [34].

#### The health care system of Pakistan

National public health is a recent innovation in Pakistan. National health planning began with the Second Five-Year Plan (1960–1965) and continued through the Eighth Five-Year Plan (1993–1998). In addition to public- and private-sector biomedicine, there are indigenous forms of treatments. Some manufactured remedies are also available in certain pharmacies. Homeopathy is also taught and practiced in Pakistan. Prophetic healing is based largely on Islamic tradition pertaining to hygiene and moral and physical health; simple treatments are used, such as honey, a few herbs and prayer. Some religious conservatives argue that reliance on anything but prayer suggests lack of faith, while others point out that the Prophet Muhammad remarked that Allah has provided a cure for every disease other than death and old age [35].

The Ministry of Health is responsible for all matters concerning national planning and coordination in the field of health. The Drugs Control Organization is a subsidiary of the Ministry of Health. It has been facilitating local pharmaceutical units and drug importers in registration and licensing and making their participation possible in various events organized worldwide [36]. Under the Pakistani Constitution, the federal government is responsible for planning and formulating national health policies; provincial governments are responsible for implementation.

The private sector serves nearly 70% of the population, whereas the public sector comprises more than 10 000 health facilities, ranging from basic health units (BHUs) to tertiary referral centers. The BHU cover around 10 000 people, whereas the larger rural health centers (RHCs) cover around 30 000 to 450 000 people. In Pakistan, primary health centre (PHC) units comprise both BHUs and RHCs. The Tehsil Headquarters Hospital (THQ) covers the population at sub district level, whereas District Headquarters Hospital serves at district level as its name suggests [37].

The health system of any country depends primarily on the human resources available. In the case of Pakistan, there is a lack of a clear, long-term vision for human resource development: the federal Ministry of Health and the provincial departments of health do not have units responsible for this important health system function. The health information system is fragmented. Each vertical programme has more or less its own information system and none covers the private health sector. There is no organized system of disease surveillance and there is limited capacity to use information for decisions. The overall capacity to undertake health policy and system research is deficient [38].

As the population is growing and there are issues of poor housing, lack of exercise, pollution, improper diet and lack of health education, diseases are rampant. The health care system in Pakistan has been confronted with problems of inequity, scarcity of resources, inefficient and untrained human resources, gender insensitivity and structural mismanagement [39]. Pakistan is facing a very precarious economic situation and there is a need of innovative health reform [40].

Political instability has caused change in the government, thus resulting in changes in health policy. Till now, health policies have not been given enough time for proper implementation in the country [41]. The low priority given to the health sector by the military regimes has resulted in a persistent contrast between reasonable economic growth and government expenditure on health[42].

The pharmacy profession in PakistanAt the time of independence – 1947 – there was no institution offering pharmacy education in Pakistan. In 1948, the University of Punjab was the first institution to start a pharmacy department; in 1964 a Department of Pharmacy was established at the University of Karachi.

The pharmacy programme was initiated as a three-year baccalaureate programme, and then in 1978–1979, it was lengthened to a four-year programme. At that time, the pharmacy curriculum was directed mainly towards production of pharmaceuticals, which helped provide the pharmaceutical industry with well-qualified and skilled human resources, but there was no consideration of the public health role of the pharmacist [24].

During recent years, in most of the public-sector hospitals, small numbers of pharmacists were appointed; their role was limited to drug delivery, procurement and inventory control. There was a lack of pharmacy services in the hospitals and community pharmacies because of the isolation and lack of recognition of pharmacists as health care professionals. The lack of trained personnel and the resulting lack of contact of pharmacists with the public are also among the main contributing factors towards the lack of recognition of the pharmacy profession.

In 2003, the Doctor of Pharmacy (Pharm.D) began to be offered as a five-year professional degree programme in Pakistan, focused mainly towards the clinical aspects of the pharmacy profession. Some 2587 pharmacists have graduated every year. With the current population, this number is not sufficient to provide optimal health care delivery [16].

There are a total of 28 pharmacy institutions in the country [43]. The Pharmacy Council of Pakistan was established under the provision of the Pharmacy Act of 1967. It regulates the practice and education of pharmacists in the country [44]. It is also responsible for registration of pharmacy graduates and issuing the license permitting them to practice in the country. Registration activity is decentralized and the regional pharmacy councils (sub bodies) under the Pharmacy Council of Pakistan are responsible for controlling and registering pharmacists in their respective provinces.

It has been estimated that around 8102 pharmacists are present in Pakistan, of whom 2836 work in the public sector and 5023 in private settings, while 243 work in private, non-profit-making organizations [38]. Among the total number of pharmacists in Pakistan, approximately 55% are engaged in the production of pharmaceuticals – 15% of them working at the federal and provincial drug control authority and hospital pharmacy level – with another 15% in sales and marketing of pharmaceuticals, 10% in community pharmacy, and the rest 5% in teaching and research [44].

Although elsewhere in the world the role of pharmacists is recognized in community pharmacies, hospital and drug regulatory authorities, the health care system of Pakistan has yet to recognize this role [45]. There are several reasons for the lack of recognition of the pharmacy profession in Pakistan, such as the lack of pharmacists in public health services and the lack of pharmacists in community pharmacies [46], which leads to the lack of community-pharmacist interaction.

The lack of recognition by other health professionals of the pharmacist's role in the health care system is due to their lack of interaction with pharmacists, as most of the pharmacy institutions in Pakistan exist without an attached hospital where pharmacy students can acquire basic clinical knowledge. To overcome this problem, it has been suggested that existing pharmacy residency programmes or specialized internships in hospitals after completion of the five-year coursework should be extended from six months to one year [47], and it should be made compulsory, with a stipend. Besides that final year, Pharm-D students must be involved in extensive clerkships in the hospitals to improve their skills as clinical pharmacists, as this will be important [48]to meet the expectations and needs of the society.

## Conclusion

The current era of globalization has witnessed evolution in the professions of the health sector, especially in pharmacy. Whereas previously the pharmacist worldwide was seen as responsible primarily for manufacturing and supplying medicines, today the pharmacist's role has evolved towards a clinical orientation. The profession is still under continuous transition. With change in the health demands, pharmacists have a further role to play in patient care.

The precise role of a pharmacist in the health setting is altering and varies significantly from country to country. In contrast to the developed world, pharmacists in developing countries are not fully executing their potential role. They are still struggling for the recognition of their role that can help improve the health care system.

Along with lack of human resources, the profession seriously lacks government interest in Pakistan. Access to and appropriate use of medicine is among the major health sector problems in most of the developing countries. The health care system without pharmacists is unable to cope effectively with most medicine-related issues. Thus, involvement of skillful and authoritative pharmacists in therapeutic procedures is necessary to improve appropriate use of medicines, eliminate medication errors, make proper use of the medicine budget by efficient management (to ensure maximum access) and ensure the implementation of National Essential Medicine List (NEML).

Legal reform is needed to achieve the health objectives of the nation to contribute towards attainment of the global Millennium Development Goals (MDG) and to achieve acceptance of the pharmacy profession as an integral part of a well-structured health care system.

## Competing interests

The authors declare that they have no competing interests.

## Authors' contributions

SA conceived the paper, drafted the outline and wrote the draft of the article. IM reviewed and edited the manuscript. MAH reviewed and contributed to the situation analysis in developing countries. MI, MA and AAS reviewed the manuscript and provided their valuable comments to improve it. MAH contributed to the reference search and read and approved the final manuscript.

## References

[B1] WHO (1946). Preamble to the Constitution of the World Health Organization as adopted by the International Health Conference. International Health Conference, New York, 19–22 June, 1946.

[B2] Anderson S (2002). The state of the worlds pharmacy: a portrait of the pharmacy profession. Journal of Interprofessional Care.

[B3] Hadzovic S (1997). Pharmacy and great contribution of Arab-Islamic science to its development. Med Arh.

[B4] Worley MM, Schommer JC, Brown LM, Hadsall RS, Ranelli PL, Stratton TP, Uden DL (2007). Pharmacists' and patients' roles in the pharmacist-patient relationship: Are pharmacists and patients reading from the same relationship script?. Research in Social and Administrative Pharmacy.

[B5] New tool to enhance role of pharmacists in health care. http://www.who.int/mediacentre/news/new/2006/nw05/en/index.html.

[B6] Hepler C, Strand L (1990). Opportunities and responsibilities in pharmaceutical care. J Hosp Pharm.

[B7] Farris KB, Llimos FF, Benrimoj S (2005). Pharmaceutical Care in Community Pharmacies: Practice and Research from Around the World. Ann Pharmacother.

[B8] Rovers JP, Currie JD, Hagel HP, McDonough RP, Sobotka JL (2003). A practical guide to pharmaceutical care.

[B9] Smith F (2004). Community pharmacy in Ghana: enhancing the contribution to primary health care. Health Policy Plan.

[B10] Jesson J, Bissell P (2006). Public health and pharmacy: A critical review. Critical Public Health.

[B11] Gilbert L (2001). To Diagnose, Prescribe and Dispense: Whose Right Is It? The Ongoing Struggle Between Pharmacy and Medicine in South Africa. Current Sociology.

[B12] Mil V (1999). Pharmaceutical care the future of pharmacy. http://dissertations.ub.rug.nl/FILES/faculties/science/2000/j.w.f.van.mil/titlecon.pdf.

[B13] SouthAfrican PC (2007). The role of pharmacist in promoting a healthy lifestyle. Pharmaciae – Official publication of the South African Pharmacy Council.

[B14] New tool to enhance role of pharmacists in health care. http://www.who.int/mediacentre/news/new/2006/nw05/en/index.html.

[B15] Zammit D (2003). How to make ethical decisions. The Pharmaceutical Journal.

[B16] Pharmacy Education and Healthcare. http://www.gcu.edu.pk/Library/NI_Feb07.htm.

[B17] Sing WS (2001). Pharmacy practice in Malaysia. Malaysian Journal of Pharmacy.

[B18] MoH (2008). Malaysia Health Statistic: Number of Pharmacist and Ratio. http://micpohling.wordpress.com/2008/03/08/malaysia-health-statistic-number-of-pharmacist-and-ratio/.

[B19] Ho DN (2008). Pharmacists may win 20-year battle.

[B20] Razak DA (2008). Really, health is but just a business.

[B21] Frances OD, Felicity S, Rita S (2008). Addressing the workforce crisis: the professional aspirations of pharmacy students in Ghana. Pharm World Sci.

[B22] Harding G (2001). Pharmacy Practice.

[B23] Smith F (2001). Pharmacy Practice.

[B24] Goel P, Ross-Degnan D, Berman P, Soumerai S (1996). Retail pharmacies in developing countries: A behavior and intervention framework. Soc Sci Med.

[B25] Good Pharmacy pratice in developing countries. http://www.fip.org/files/fip/Statements/latest/Dossier%20003%20total.PDF.

[B26] Gould W, Taylor N, Horwitz S, Barry M (1991). Misinformation about medications in rural Ghana. Soc Sci Med.

[B27] Pakistan. http://www.imshealthcanada.com/web/channel/0,3147,64639575_63872702_76856130,00.html.

[B28] World Atlas .com. http://graphicmaps.com/webimage/countrys/asia/pk.htm.

[B29] Chapman N, Bennett J, Khan T, Vickery C, Malik S, Ahmed I (2008). Evaluation of DFID, country programmes country study Pakistan.

[B30] Pakistan Fact Sheet. http://www.iptu.co.uk/content/trade_cluster_info/pakistan/factsheet_apr08.pdf.

[B31] Key Facts. http://www.dfid.gov.uk/Where-we-work/Asia-South/Pakistan/Key-facts/.

[B32] WorldBank (2007). Pakistan: Growth Drives Poverty Reduction. World Bank Report.

[B33] Pakistan Country Overview 2006. http://web.worldbank.org/WBSITE/EXTERNAL/COUNTRIES/SOUTHASIAEXT/PAKISTANEXTN/0,,menuPK:293057~pagePK:141159~piPK:141110~theSitePK:293052,00.html.

[B34] Pakistan Country Assistance Strategy. http://siteresources.worldbank.org/PAKISTANEXTN/Resources/CAS/PSE-Context.pdf.

[B35] Pakistan Health Care Policies and Developments. http://www.photius.com/countries/pakistan/society/pakistan_society_health_care_policies~10390.html.

[B36] Ministry Of Health. http://www.dcomoh.gov.pk/.

[B37] Ghaffar A, Kazi BM, Salman M (1999). An Overview of the Health Care System in Pakistan. Journal of Public Health Medicine.

[B38] Report of the Health System Review Mission – Pakistan. http://gis.emro.who.int/HealthSystemObservatory/PDF/HealthSystemReviewMissionReports/Pak%20HSD%20Mission%20Report%20Draft%20Ver%201%200%20March%2030%202007.pdf.

[B39] Babar ZU (2006). Pakistan National University of Pharmaceutical Sciences. American Journal Of Pharmaceutical Education.

[B40] Islam A (2002). Health Sector Reform in Pakistan: Why it is needed?. J Pak Med Assoc.

[B41] Khan MM, Heuvel WVd (2007). The impact of political context upon the health policy process in Pakistan. Public Health.

[B42] Government of Pakistan, Finance Division, Wing EA (2006). Economic survey of Pakistan, 2005–2006. Islamabad.

[B43] Healh Professional Education. http://www.emro.who.int/hped/colleges.asp?colg=Pharmacy.

[B44] Ahsan N (2005). Pharmacy Education and Pharmacy Council of Pakistan. Pakistan Drug Update Islamabad.

[B45] Babar ZU (2007). Going Back in Time. Chowk.

[B46] Khan AA (2009). Re-defined role of pharmacist in public sector hospitals of Pakistan. 15th International Pharmacy Conference and Exhibition.

[B47] Ghayur MN (2008). Pharmacy Education in Developing Countries: Need for a Change. Am J Pharm Educ.

[B48] Ahmad M, Durr-e-Shahwar, Madiha, Madni A, Usman M, Asghar W (2009). Economical and clinical impact of pharmacists' participation in patient care. 15th International Pharmacy Conference and Exhibition.

